# Does ethnic-racial identity modify the effects of racism on the social and emotional wellbeing of Aboriginal Australian children?

**DOI:** 10.1371/journal.pone.0220744

**Published:** 2019-08-07

**Authors:** Davi M. Macedo, Lisa G. Smithers, Rachel M. Roberts, Dandara G. Haag, Yin Paradies, Lisa M. Jamieson

**Affiliations:** 1 Indigenous Oral Health Unit, Adelaide Dental School, The University of Adelaide, Adelaide, Australia; 2 BetterStart Child Health and Development Research Group, School of Public Health, The University of Adelaide, Adelaide, Australia; 3 School of Psychology, The University of Adelaide, Adelaide, Australia; 4 Alfred Deakin Institute for Citizenship and Globalisation, Deakin University, Melbourne, Australia; University of New South Wales, AUSTRALIA

## Abstract

**Objectives:**

This study investigates the protective role of ethnic-racial identity (ERI) affirmation on the longitudinal association between racism and Aboriginal Australian children’s social and emotional well-being (SEWB).

**Methods:**

408 children from the K-Cohort of the Longitudinal Study of Indigenous Children were included in the analysis. Data were collected through questionnaire-guided interviews at 7–10 and 9–12 years of age. Children’s racism experience, SEWB (Strengths and Difficulties Questionnaire), and confounding were reported by caregivers. ERI was reported by children and dichotomized into high versus low. Generalized linear models with log-Poisson links and robust errors were used to estimate adjusted Risk Ratios (RR_a_) for the effect of racism on SEWB domains. Effect-measure modification analysis was used to verify differences on effect sizes per strata of ERI affirmation. The presence of modification was indicated by the Relative Excess Risk due to Interaction (RERI).

**Results:**

Slightly above half (51.4%) of the children presented high ERI affirmation. Children exposed to racism and with low ERI affirmation were at increased risk of hyperactive behavior (RR_a_ 2.53, 95% CI 1.17, 5.48), conduct problems (RR_a_ 2.35, 95% CI 1.07, 5.15), and total difficulties (RR_a_ 1.73, 95% CI 0.84, 3.55). Positive RERIs indicated the joint effects of racism and low ERI affirmation surpassed the sum of their separate effects in these domains. Children with high ERI affirmation were at increased risk of peer problems (RR_a_ 1.66, 95% CI 0.78, 3.52).

**Conclusions:**

These findings suggest that ERI may mitigate the risk of poor SEWB due to racism. Fostering affirmative ERI can be an important strategy in promoting resilience in Aboriginal Australian children.

## Introduction

Racism can be defined as a system of practices, attitudes and beliefs that assumes the inferiority of certain ethnic-racial groups in relation to others which are considered superior, sustaining an unequal and avoidable distribution of resources based on ethnic-racial membership [1, 2]. The maintenance of oppression occurs structurally (differences in education, income, health, and political representation), interpersonally (direct experiences of race-based discrimination), and individually (internalization of societal messages of inferiority by ethnic-racial minorities or superiority by ethnic-racial majorities) [1, 2]. In Australia, racism against Aboriginal people is a pervasive social problem and a public health issue [3], with significant economic costs [4]. Disadvantages are observed in levels of education, income, employment opportunities, health indicators, and life-expectancy relative to the non-Aboriginal population [5].

Experiences of interpersonal race-based discrimination are reported by Aboriginal Australians across the lifespan, starting from childhood [6] and continuing through to older years [7]. Interpersonal racism negatively compromises daily routines and impacts Aboriginal Australians’ health and well-being. It is established that racism is a normative experience among ethnic-racial minority youths and its effects can be observed from childhood [8]. Among children racism is shown to be associated with higher levels of anxiety, depression, aggression, conduct problems, social and emotional difficulties, and also lower levels of self-esteem, psychological well-being, and quality of life [9]. Research with Aboriginal Australian children and youth shows similar associations, with racism being linked to depressive symptoms, emotional and behavioral difficulties, and poor overall mental health [3].

Identification of protective factors and early intervention is relevant to preventing negative outcomes from escalating and promoting positive development among children experiencing adversity [10]. The present study focuses on the role of ethnic-racial identity as a protective factor against the detrimental effects of racism on child well-being. ERI can be defined as one’s beliefs and attitudes about belonging to a given ethnic-racial group [11, 12]. Ethnic and racial identity have been previously theoretically distinguished as separate terms. Ethnic identity has been used to refer to perceptions relative to one’s cultural beliefs, values, and behaviors [13]. Racial identity is claimed to be the construct of interest when researchers are interested in identity formation processes in a racialized society, being generally focused on responses and attitudes to racism [13, 14]. ERI in this context refers to experiences that are not uniquely ethnic or racial, but that interact with race and ethnicity in a dynamic process [11, 12]. This is the orientation of the Ethnic and Racial Identity in the 21^st^ Century Study Group, formed to provide guidance on future research in the area [11]. The justification for adopting the ERI meta-construct is that race and ethnicity might be inseparable for some ethnic-racial groups due to the interplay of heritages, phenotypes, racial socialization experiences, and exposure to racism [11, 12].

A sense of ERI is as a multidimensional concept [15] that can be observed from early age [16]. It involves perceptions and attitudes about group belonging, exploration of cultural practices and behaviors, understanding of stereotypes held by in-group and out-group members, and the levels of commitment and attachment to one’s ethnic racial group [11, 12, 16]. The degree of exploration of one’s ERI and how they feel about such identity influences how youths experience and respond to racism. The link between racism and ERI helps to elucidate how ethnic-racial minority youths reconcile the need for a positive and healthy identity in a context that communicate their marginalized status [17].

Phinney’s model might be one of the most important proposals of ERI development, focusing on exploration of cultural practices and commitment to ERI [11, 15]. The author’s Multi-group Ethnic Identity Measure (MEIM) grounds several studies in adolescence and adulthood [11, 18]. This reflects the increase of complexity of ERI exploration during these developmental periods and is shown in the level of complexity in the phrasing of the items used (e.g, “I understand pretty well what my ethnic group membership means to me”) [15]. Critics of these approach suggest that the ERI attitudinal component might be distinct from the level of exploration of cultural practices and commitment to one’s ERI [19]. Orientation for future research is that these domains be assessed separately [11, 12, 19] Though recognizing the relevance of understanding ERI increased complexity over time, the present study focuses on ERI content.

As the understanding of one-self as a member of a social group, the positive and negative affects attached to membership are especially relevant to the perception of one’s ERI [20]. Scholars suggest that a sense of belonging and positive affect towards the ethnic-racial in-group can be considered one of ERI’s most important aspects [15, 20, 21]. This perspective is in accordance with the Social Identity Theory rationale, according to which positive attitudes about a given social identity contributes to increased self-esteem, well-being and assuring a positive self-concept in the present of threat to one’s social group [22,23]. In a developmental perspective, in middle childhood children possess the levels of cognitive maturation (e.g, social comparison and perspective-taking abilities) and socialization that allow for the assessment of in-group and out-group attitudes [12]. A strong sense of belonging and pride in regard to the Aboriginal culture is also emphasized as a central determinant of Aboriginal children’s well-being and resilience by Aboriginal Australians [24, 25]. For the purposes of the present study, positive feelings towards ERI is understood as ERI affirmation [11, 19].

ERI has been internationally identified as an important determinant of positive Indigenous youth mental health and development. Canadian First Nation children in middle childhood are shown to possess a sense of ERI from age 6, with positive in-group evaluation and relevance of ERI for self-concept increasing with age [16, 21]. In the Australian context, Aboriginal children in middle childhood are shown to possess a sense of ERI with an important focus on exploration of cultural values and beliefs [26, 27]. Among First Nation Canadian youth, a clear sense about one’s ERI was shown to contribute to the establishment of a consistent and defined sense of self, positive self-esteem, and psychological well-being [28], being also associated with self-efficacy, school connectedness, and life satisfaction [29]. Among American Indian youth, ERI is found to be inversely associated with depression, anxiety, and externalizing behaviors and to have a positive relationship with psychosocial functioning [30, 31]. Despite evidence of the association between ERI and positive development in childhood [32], most studies are conducted with children in the late years of childhood and adolescence [29, 32, 33].

The protective role of ERI on the detrimental effects of racism is also acknowledged in the literature [34, 35]. Positive regard and commitment towards ERI is shown to buffer the association between racism and depressive symptoms among African American adults and adolescents [34, 36]. At a physiological level, African American men and women to whom ERI was a central component of self were shown to present low stress-response system reactivity during discriminatory encounters. Facilitated protective inflammatory processes during episode recovery was also observed [37]. Once more, most of the research is conducted in adulthood and adolescence [34, 36]. Nonetheless, there is initial evidence of the protective role of ERI for the effects of racism in childhood. A strong sense of ERI was found to buffer the effects of racism on children’s internalizing and externalizing symptomatology among 7–8 years old from different racial-ethnic backgrounds (e.g., African-American, Latin-American, multi-ethnic racial). Commitment to ERI was especially salient to mitigate racism effects on internalizing symptoms, suggesting the importance of ERI to self-concept related psychological processes in childhood [35].

Despite initial evidence, further research on how ERI helps to promote children’s positive development in the context of racial discrimination is still necessary [16, 35]. The present study aims to investigate if positive ERI can modify the association between racism and Aboriginal children’s social and emotional wellbeing (SEWB). As most studies on the effects of racism are still cross-sectional [9], we sought to investigate associations and the protective role of ERI in a longitudinal study. In other words, we investigated if the longitudinal effect of racism on different domains of child SEWB (hyperactive behavior, peer problems, conduct problems, and emotional difficulties) differs among children with different levels of ERI affirmation. Our main hypothesis is that the effects of racism will be smaller among children with high levels of ERI affirmation in all domains of SEWB analyzed.

We propose effect-measure modification analysis as an innovative approach to this area of research due to its applicability to public health research and policy formulation. The presentation of risk ratios per strata of the exposure and the effect-modifier provides a more transparent analysis as we disclose effect sizes under each condition [38]. Previous research results are based on structural equation models [35]. Under many circumstances, the assumptions underpinning structural equation modelling, including linearity and no confounding, are untenable [39]. These assumptions must hold for the relationships between all exposure, outcomes, confounding, mediator, and modifier variables in the model [39]. Effect-measure modification in the counterfactual framework makes assumptions that hold for the exposure and outcome relationship only. Additionally, to the best of our knowledge no research has investigated the relationship between racism, ERI, and well-being among Aboriginal Australian children. We aim to add to the evidence of the role of ERI for the well-being and development of Aboriginal Australians. We are limited to the data available, which is subject to small samples in some strata. We present the results in a way that will allow future inclusion in a meta-analysis, should more data become available in the future.

## Methods

### Study design

Data were from the Longitudinal Study of Indigenous Children, a national survey funded by the Australian Government Department of Social Services to collect information on determinants of Aboriginal Australian and Torres Strait Islander children’s health and development. The study is a pioneer in collecting information on experiences of racism and resilience factors among Aboriginal children at a national level [40]. Its accelerated cross-sequential design involves two cohorts of children: The Baby Cohort (B Cohort) and the Child Cohort (K Cohort). Data used in this study was from the K Cohort, which includes children who were 3.5 to 5 years old at wave 1. The following waves occurred annually. Access to data from waves 1 to 9 is currently available upon application and a signed deed of license from legislated bodies. More information is available at the Australian Government Department of Social Services website [40].

### Data collection procedures

The first stage of sampling involved the selection of 11 sites—including remote communities to capital cities—representative of the socioeconomic and community environments where Aboriginal children lived [40]. A non-representative purposive sample was recruited using addresses provided by Centrelink and Medicare Australia. Centrelink is part of the Australian Department of Human Services, delivering social security payments for people who are unemployed or unable to work [41]. Medicare Australia provides benefits to assist with the costs of health services [42]. Eligible participants were approached, and signed consent obtained. Neither the probability of being selected for the study among the total Australian Indigenous population, nor the selection of families within each study site, occurred through a randomized process. Details about the interviewing process across waves are explained elsewhere [40].

Information was obtained through questionnaire-guided interviews conducted by trained Aboriginal and Torres Strait Islander Research Administration officers. The child’s main caregiver, child’s secondary caregiver, child’s teacher, and the study child were interviewed. Written consent was provided by all informants and authorization for data collection with the study child was signed by caregivers. Participants were fully informed that they could withdraw from the study at any time. Each child received a unique identifier that remained constant in all the subsequent waves for all interviews related to that child. Ethical approval was obtained from the Australian Government Department of Health Departmental Ethics Committee. State/territory and or regional ethics clearance from the LSIC locations Human Research Ethics Committees were also obtained [40].

### Participants

Information on ERI was assessed solely at wave 8 among children in the K-Cohort. All the children with available information on ERI (n = 408) were included in the analysis. Information on racism and all confounding variables was collected in wave 6 (2013), when the children were aged 7 to 10 years. Data on SEWB was collected two years later at wave 8. The child’s main caregiver provided the data on exposure, outcomes, and confounding variables. The effect-modifier (ERI) was collected through child self-report. The children were aged 9 to 12 years (wave 8).

### Measures

#### Racism (exposure)

Caregivers answered the question “Has study child been bullied or treated unfairly at school because he/she is Aboriginal and/or Torres Strait Islander?”. Answer options were “Yes, bullied (kids being mean to him/her)”, “Yes, treated unfairly (adults being mean to him/her)”, “Yes, both bullied and treated unfairly”, and “No”. Answers were dichotomized in “Yes” and “No” for analysis purposes.

#### Child social and emotional well-being (outcome)

The Strengths and Difficulties Questionnaire (SDQ) was used to assess emotional and behavioral difficulties associated with higher risk for the onset of future psychological disorders. The measure is valid for use among 4 to 17 years old and is comprised of 25 questions that assesses child difficulties and potentialities in five domains: emotional symptoms, hyperactive behavior, conduct problems, peer problems, and pro-social behavior [43]. Only the first four domains were included in the analysis as to focus on potential difficulties on SEWB. The score range is from 0 to 10, with higher scores indicating greater difficulties. A SDQ Total score for emotional and behavioral problems was also used, ranging from 0 to 40. All scores were dichotomized based on cut-off points for above the threshold for emotional and behavioral difficulties. Scores equal or above 5 were considered risk for emotional symptoms, equal or above 7 to hyperactivity, equal or above 4 for both conduct and peer problems, and equal or above 14 for the total SDQ score [44].

#### ERI affirmation (potential effect-modifier)

The study child answered a set of four questions regarding ERI at school, all with a 6-point Likert Scale ranging from “Yes (Always)”, “Yes (Most of the time)”, “Sometimes (Fair bit)”, “Sometimes (Little bit)”, “No (Not much)”, “No (Never)”. The questions were: 1) “I feel good about being Aboriginal and/or Torres Strait Islander in class”; 2) “I want to share (tell others) things about being Aboriginal and/or Torres Strait Islander in class”; 3) “I feel safe about being Aboriginal and/or Torres Strait Islander in class”; and 4) “I like people to know I am Aboriginal and/or Torres Strait Islander in class”. Item’s reliability was considered satisfactory (Cronbach’s Alpha: 0.71). A single dichotomized variable was generated for different levels of ERI affirmation. Children who answered to “Yes (Always)” and “Yes (Most of the time)” to all four questions were included in the “High ERI affirmation” category.

#### Confounding variables

Confounders were conceptualized as variables that share a causal association with both exposures and outcomes [45]. The confounders included in our adjusted models were study child sex, age, and dominant language, main caregiver level of education, family Index of Relative Indigenous Socioeconomic Outcomes (IRISEO), and Level of Relative Isolation (LORI) [9, 46, 47]. The Study Child dominant language was derived from the question “What language (s) is Study Child learning?”, collected at wave 8. Response options were English, foreign languages, and Aboriginal languages (e.g. Alyawarr, Pitjantjatjara, Yorta-Yorta). Answers were categorized in “Equally fluent in English and in an Indigenous language”, “Dominant in an Indigenous language” and “Dominant in English”.

The main caregiver highest level of education attainment was collected at waves 2 and 3 and response options ranged from “Never attended school” to “Post-graduate degree”. Responses were reclassified in four categories: “Year ten or below of high school”, “Year 11 or 12 of High School”, “Post school certificate/Advanced diploma”, “Graduate degree or above”. The IRISEO is a measure of community level socioeconomic advantage linked to the LSIC data set. It is calculated specifically for Indigenous people, based on income, employment, education and housing indexes from the Australian 2006 Census. The IRISEO is measured in deciles, ranging from most disadvantaged (1) to most advantaged (10) [48]. Family LORI is a measure of remoteness/isolation based on the Accessibility/Remoteness index of Australia. It is calculated based on relative distance to service centers. The LORI categories range from “no isolation”, which corresponds to metropolitan areas to “low isolation”, “moderate isolation”, “high isolation” and “extreme isolation”[49].

### Analysis

Analyses were conducted in Stata 14. Multiple imputation with chained equations (MICE) was used to address potential bias due to missing data. Twenty data sets were generated from imputation models using all the variables tested in the association models. MICE analyses were based on the assumption of missingness at random conditional on the observed data [50]. Imputed values were generated for children’s experience of racism (missing = 10), main caregiver level of education (missing = 7), study child dominant language (missing = 18), child ERI affirmation (missing = 26), and the multi-level effect-modifier variable (missing = 35), explained below. All other variables were respondent sample complete cases. After imputation, all analyses were conducted based on a final sample of 408 children.

Descriptive analyses were performed to characterize the sample. We present prevalence estimates with confidence intervals as the number of participants varies among the imputed data sets, as is always the case when using imputed data. Generalized linear models with a log-Poisson link and robust errors were used to calculate Risk Ratios (RR) as unadjusted effect estimates of racism on the four individual domains of child SEWB and on the SDQ total difficulties score. Next, adjusted RR (RR_a_) were obtained after the inclusion of confounding variables in the analysis, as above. Risk Ratios were calculated rather than odds ratio as the latter tends to be inflated when the outcome is not rare. Risk Ratios are also more relevant for public health purposes as it considers the proportion of the outcome among the entire population at risk [51]. For the effect-measure modification analysis, RR_a_ were obtained for the different stratum of the exposure (racism) and the effect-modifier (ERI affirmation) [38, 52]. A categorical variable with four levels was entered into the adjusted model as exposure to represent the following stratum:

- non-exposure to racism and high ERI affirmation (reference group);
- non-exposure to racism and low ERI affirmation;
- exposure to racism and high ERI affirmation; and
- exposure to racism and low ERI affirmation.

Subsequently, the RR_a_ for the effects of racism on the SEWB domains within strata of ERI affirmation was estimated separately [38]. Effect-measure modification occurs when the effect of a main exposure differs across levels of a second exposure [53]. We present an effect-measure modification analysis because our main interest is the protective role of ERI affirmation on the effects of racism. Although it is important to reduce racism against Aboriginal people in society [54], we aim to verify if ERI can assist Australian Aboriginal children to navigate through racist discrimination experiences whilst initiatives to reduce societal levels of racism are designed and implemented. The effect-measure modification is presented in the risk-difference scale. The risk-difference scale estimates if the effect of the interaction of both exposures surpasses the sum of the two separate effects added together. It allows us to identify differences in the levels of the outcome per strata of the effect modifier [53]. In the case of the present study, it enables us to identify the reduction on the detrimental effect of racism on child SEWB when child ERI affirmation is high.

The effect-measure modification on the additive scale was obtained by the calculation of a Relative Excess Risk due to Interaction (RERI). The RERI was calculated through the formula RR(d)–RR(b)–RR(c) + RR(a), where a, b, c, and d represent the levels of the exposure and effect-modifier presented above. A RERI higher than 0 suggests that the effects of the two exposures operating together is higher than that of each added together (the effect measure modification is positive). In other words, it suggests that the effect of racism interacting with low ERI affirmation is higher than the sum of the independent effects of racism and low ERI affirmation. A RERI of 0 suggests no effect-measure modification is present, whilst a negative value suggest the effect-measure modification operates in a negative direction [35]. Rather than interpreting the RERI size, we interpret the RERI in terms of the direction in which the effect-measure modification estimated occurs, as recommended by Knol & VanderWeele [38].

In the present study, we focus on the size of the effects and compare effects across strata of the variables of interest (racism exposure and level of ERI affirmation), as recommended by the American Statistical Association and the American Psychological Association [55, 56]. P-values and confidence intervals are shown to be highly dependent on the sample size, which can bias conclusions driven by interpretation of statistical significance [57, 58, 59]. Confidence intervals were interpreted as indicators of the precision of the effect estimate, and not as having a 95% probability of including the true effect size in the population, as commonly misinterpreted [59, 60]. Comparisons of effect-sizes per stratum of exposure provided useful information on the risk differences among groups and on the verification of the protective role of ERI affirmation for children exposed to racism.

## Results

The mean child age when information on racism was collected was 8.5 years (SD 0.5) and the sample consisted of 51% girls and 49% boys. Approximately 15% of children were reported to have experienced racism at school and between 15% and 22% of the children presented difficulties in any of the investigated SEWB domains. Slightly above half of the children (51.4%) presented high ERI affirmation. Further information is presented in Table 1.

**Table 1:** Means and frequencies (95% CI) of exposure, outcomes, effect-modifier and confounding variables.

**Means (Standard Error)**	
**Child Age (years)**	8.5 (0.03)
**Family IRISEO**^**a**^	5.8 (0.11)
**Prevalence (95% CI)**	
**Racism**^**b**^	
Yes	15.6 (12.0, 19.2)
No	84.3 (81.0, 88.0)
**Child social and emotional wellbeing**^**c**^	
High SDQ Emotional difficulties	15.0 (11.2, 18.41
High SDQ Hyperactive behaviour	18.0 (14.0, 21.6)
High SDQ Conduct problems	18.0 (14.1, 21.6)
High SDQ Peer problems	20.3 (16.4, 24.2)
High SDQ Total difficulties score	22.5 (18.5, 26.6)
**Ethnic-racial identity**^**d**^	
High	51.4 (46.0, 56.5)
Low	48.6 (43.6, 53.6)
**Sex**	
Male	49.0 (44.0, 54.0)
Female	51.0 (46.0, 56.0)
**Child dominant language**	
Equally fluent—English and Indigenous language	5.8 (3.5, 8.1)
Indigenous language	4.6 (2.5, 6.8)
English	89.5 (86.5, 92.5)
**Main caregiver level of education**	
Year 10 of High School or below	31.6 (27.0, 36.2)
Year 11 or 12 of High School	26.0 (22.0, 30.4)
Post School certificate or Advanced diploma	32.2 (28.0, 37.0)
Graduate degree or above	10.0 (7.0, 13.0)
**Family Level of Relative Isolation (LORI)**^**e**^	
None	28.6 (24.2, 33.0)
Low	54.0 (49.0, 59.0)
Moderate	9.8 (7.0, 12.7)
High/Extreme	7.5 (5.0, 10.0)

^a^ Index of Relative Indigenous Socioeconomic Outcomes (IRISEO) is a measure of community level socioeconomic advantage calculated specifically for Indigenous people, based on income, employment, education and housing indexes from the Australian 2006 Census. The IRISEO is measured in deciles, ranging from most disadvantaged (1) to most advantaged (10).

^b^ Child experience of racism was caregiver-informed. Answers to the question “Has study child been bullied or treated unfairly at school because he/she is Aboriginal and/or Torres Strait Islander?” were dichotomized into “Yes” or “No”.

^c^ Child social and emotional wellbeing was reported by caregivers’ response to the Strengths and Difficulties Questionnaire. The measure provides domain-specific indicators and a total indicator of emotional and behavioural difficulties.

^d^ Child report of Ethnic-Racial Identity affirmation was assessed by the questions: 1) “I feel good about being Aboriginal and/or Torres Strait Islander in class”; 2) “I want to share (tell others) things about being Aboriginal and/or Torres Strait Islander in class”; 3) “I feel safe about being Aboriginal and/or Torres Strait Islander in class”; and 4) “I like people to know I am Aboriginal and/or Torres Strait Islander in class”. Children who answered “Yes (Always)” and “Yes (Most of the time)” to all questions were included in the “High ERI affirmation” category.

^e^ Family LORI is a measure of remoteness/isolation based on the Accessibility/Remoteness Index of Australia (ARIA). It is calculated based on relative distance to service centres. The LORI categories range from “no isolation”, which corresponds to metropolitan areas to “low isolation”, “moderate isolation”, “high isolation” and “extreme isolation”.

The adjusted risk ratios for the longitudinal effects of racism on child SEWB (Table 2) show that the point estimates were more prominent for hyperactive behavior (RR 1.65, 95% CI 0.65, 2.06), and peer problems (RR 1.25, 95% CI 0.76, 2.07), though the 95% confidence intervals were wide. The risk of total behavioral and emotional difficulties due to exposure to racism was of 18% (RR 1.18, 95%CI 0.72, 1.94) when compared to children not exposed.

**Table 2:** Effects of racism at school on children’s social and emotional wellbeing^a^.

	Emotional Symptoms	Conduct problems	Hyperactivity	Peer problems	Total difficulties
Unadjusted Risk Ratios (95% CI)	1.18 (0.63, 2.20)	1.19 (0.70, 2.04)	1.48 (0.89, 2.46)	1.23 (0.75, 2.01)	1.12 (0.69, 1.81)
Adjusted Risk Ratios (95% CI)	1.17 (0.61, 2.26)	1.16 (0.65, 2.06)	1.65 (0.99, 2.75)	1.25 (0.76, 2.07)	1.18 (0.72, 1.94)

^a^ Child social and emotional wellbeing was caregiver-informed through the Strengths and Difficulties Questionnaire. The measure provides domain-specific indicators and a total indicator of emotional and behavioural difficulties.

The analysis of the effect-measure modification of ERI on racism effects on children’s SEWB are shown in Table 3. Among children with low ERI, there was a 43% increased risk (RR_a_ 1.43, 95%CI 0.52, 3.90) of emotional difficulties for the children who experienced racism, although the estimate is imprecise, as indicated by the wide confidence intervals. Whereas among children with high ERI, the point estimate indicates a 10% lower risk of emotional difficulties, and again, the confidence intervals were wide and we cannot reasonably rule out a null association (RR_a_ 0.90, 95% CI 0.31, 2.59). In the analysis including all children, the highest risk of having emotional problems was among children who had low ERI and were also exposed to racism (RR_a_ 1.76 (95%CI 0.66. 4.64)). This was confirmed by the RERI, where there appears to be effect measure modification on the additive scale. A RERI = 0.51 (95%CI -1.36, 2.38) suggests that the effect of racism operating together with low ERI affirmation on emotional difficulties (RR_a_ 1.76) was higher than the sum of the individual effects of racism (RR_a_1.05) and low ERI affirmation (RR_a_ 1.19).

**Table 3:** Effect-measure modification of ERI on the effects of racism on all domains of SEWB.

	Racism = No		Racism = Yes		
	N High Score /Low Score	RR_a_^a^ (95% CI)	N High Score /Low Score	RR_a_ (95% CI)	RR (95% CI) for racism within strata of ERI
**Emotional Difficulties**					
**Low ERI**	26/149	1.19 (0.65, 2.19)	6/19	1.76 (0.66, 4.64)	1.43 (0.52, 3.90)
**High ERI**	24/147	1	5/32	1.05 (0.38, 2.88)	0.90 (0.31, 2.59)
Effect-measure modification on the risk-difference scale: RERI = 0.51 (-1.36, 2.38) p = 0.59.					
**Hyperactivity**					
**Low ERI**	32/143	1.10 (0.64, 1.90)	9/16	2.53 (1.17, 5.48)	2.16 (1.00, 4.67)
**High ERI**	26/145	1	6/31	1.34 (0.54, 3.30)	1.38 (0.63, 3.00)
Effect-measure modification on the risk-difference scale: RERI = 1.08 (-0.93, 3.11) p = 0.29					
**Conduct Problems**					
**Low ERI**	35/140	1.25 (0.73, 2.13)	9/16	2.35 (1.07, 5.15)	1.76 (0.81, 3.83)
**High ERI**	26/145	1	4/33	0.70 (0.23, 2.07)	0.77 (0.31, 1.93)
Effect-measure modification on the risk-difference scale: RERI = 1.39 (-0.40, 3.20) p = 0.12					
**Peer problems**					
**Low ERI**	39/136	1.28 (0.77, 2.13)	5/20	1.19 (0.46, 3.07)	0.88 (0.33, 2.34)
**High ERI**	29/142	1	10/27	1.66 (0.78, 3.52)	1.80 (0.83, 3.90)
Effect-measure modification on the risk-difference scale: RERI = -0.75 (-2.47, 0.97) p = 0.39.					
**Total emotional and behavioural difficulties**					
**Low ERI**	37/138	0.84 (0.52, 1.36)	10/15	1.73 (0.84, 3.55)	1.94 (0.92, 4.11)
**High ERI**	41/130	1	6/31	0.74 (0.30, 1.79)	0.74 (0.30, 1.83)
Effect-measure modification on the risk-difference scale: RERI = 1.14 (-0.15, 2.44), p = 0.08.					

^a^ RR_a_ are adjusted for child age, sex, dominant language, parental education, family IRISEO, and LORI.

Consistent with the results on the emotional domain, the risks of hyperactive behavior was doubled (RR_a_ 2.16, 95%CI 1.00, 4.67), conduct problems was 76% higher (RR_a_ 1.76, 95% CI 0.81, 3.83), and total difficulties were 94% higher (RR_a_ 1.94, 95% CI 0.92, 4.11) for low ERI children who experienced racism, compared with children who did not experience racism. Among all children, the ones with low ERI exposed to racism had the higher risks for hyperactivity (RR_a_ 2.53, 95% CI 1.17, 5.48), conduct problems (RR_a_ 2.35, 95% CI 1.07, 5.15), and total difficulties (RR_a_ 1.73, 95% CI 0.84, 3.55). An effect-measure modification on the additive scale was confirmed for each of these domains, with values ranging from 1.08 to 1.39.

The opposite pattern for the previous domains was observed for peer problems. Among the children with high ERI affirmation, experiencing racism was associated with an 80% higher risk of presenting peer problems (RR_a_ 1.80, 95% CI 0.83, 3.90). Among all children, those discriminated with high ERI affirmation had the higher risk (RR_a_ 1.66, 95% CI 0.78, 3.52). A RERI of -0.75 suggests a negative effect measure modification in the additive scale. In other words, the effects of racism and low ERI affirmation (RR_a_ 1.19) was lower than the expected sum of the individual effects of racism (RR_a_ 1.66) and low ERI affirmation (RR_a_ 1.28).

## Discussion

The results demonstrate the protective role of ERI affirmation on the longitudinal effects of racism on Aboriginal Australian children SEWB. As hypothesized, our findings suggest the longitudinal association between racism and SEWB varies according to ERI affirmation. Children with low ERI affirmation whose parents reported they experienced discrimination/racism were at increased risk of poor SEWB two years later, with more prominent effects for the onset of hyperactive behavior, conduct problems, and total difficulties. A positive effect-measure modification was found for the effects on child emotional difficulties, hyperactive behavior, conduct problems, and the total difficulties score. In other words, it shows that promoting ERI affirmation can mitigate the effects of racism on these SEWB domains. Effect-measure modification is informative to identify groups in which a specific intervention might be more effective [53]. Here we use it as to demonstrate the protective role of ERI on the effects of racism on Aboriginal children SEWB.

Contrary to our hypotheses, a negative effect-measure modification was found in the peer problems domain. Analysis per strata showed a double increased risk for children in the high ERI affirmation group. This raises the question of whether Aboriginal children with higher ERI affirmation might be targeted for discriminatory peer behavior because their ERI is more salient [17]. It is also possible that peer problems arise as a result of confronting peer racial discrimination, which might be more common among children in the higher ERI affirmation group. The increase in peer problems amongst Aboriginal children with higher ERI might indicate the need to discuss multi-culturalism and racism in the school setting to reduce racially motivated conflict among students [6]. Broadly speaking, interventions to promote awareness and prevent racism against Aboriginal people in Australia are an important public health goal [61].

The results observed in the peer problem scale, however, should be interpreted with caution. Although Aboriginal caregivers stated peer relationships as important, relationships with the extended family and the Aboriginal community generally were thought to be substantially more relevant [62]. Considering the centrality of family and kinship for Aboriginal people [24], caregivers did not see having few friends as dysfunctional, provided connection with family members and the broader Aboriginal community were strong [62]. Research on the psychometric properties of the SDQ among Aboriginal Australian children suggest the peer problem scale is the least reliable, with the scale performing more poorly among children residing in remote locations [63, 64]. Removing the peer problems scale, however, did not improve the fit of the five-item original model of the instrument, suggesting the complete version of the SDQ is still valid for use with Aboriginal children [63]. Additionally, poor internal consistency of the SDQ peer problems scale have been found in other populations [65]. Future studies might address how applicable the peer problems subscale is for the understanding of SEWB among Aboriginal Australian children.

Our results are in accordance with the literature on the importance of ERI as a determinant of Aboriginal Australian children’s SEWB and its potential as a protective factor against risk exposure [25, 28, 35]. It corroborates the importance of promoting ERI affirmation and integrating Aboriginal Australians’ cultural values and beliefs for health and well-being promotion [24]. Results should be interpreted with care as the confidence intervals were wide, likely because of the small number of children in some subgroups. We believe that although larger samples might improve the precision of the estimates, our results are based on one of the best available information sources on determinants of Aboriginal children’s development. The LSIC has a large sample size, with annual follow-ups, and covers a range of geographical locations, and health and well-being indicators [40]. Thus, larger samples are not available in Australia. The LSIC is also one of the few and largest studies to include information on Indigenous experiences of racism and ERI in childhood [66, 67]. We acknowledge that the LSIC sample is not-representative of the Aboriginal children population, as a non-random purposive sampling design was used [40]. Nonetheless, representativeness is not required for estimating casual associations [68].

The ASA emphasizes that research results must be interpreted according to the study design, methods to minimize chance, bias, and confounding, and previous evidence shown in the literature [55, 69]. We believe we addressed adjustment for confounding based on available evidence of variables that can be a source of confounding bias in the effects estimated. We also used multiple imputation with chained equations to address potential non-response bias. The calculated RERI as an indication of effect-measure modification in the additive scale provided a clear comparison between the effects of a risk factor (racism) among different subpopulations. This is important information to help facilitate evidence-based public policy design and resource allocation [38]. The interpretation of statistical results followed guides of reference associations in the Statistics and Psychology fields [55, 56]. While we are concerned about the small sample sizes, this is possibly the only data available of this kind and is therefore an important contribution to the literature. Furthermore, we have presented in a way that would allow for the inclusion of the estimates in future meta-analysis, shall these variables be collected in future studies.

The literature acknowledges the tendency of ethnic-racial minorities to underestimate experiences of racism [70]. Besides, information on racism was caregiver-informed, which might translate only the racism experiences that they were aware of, again underestimating levels of racism exposure from the child’s perspective [9]. Nonetheless, self-report measures of racism are generally used in samples aged 12 or older in the context of Aboriginal Australian research [71, 72]. The LSIC research team and community stakeholders consulted possibly considered the children to be too young to respond to such a sensitive topic. Our results, however, are in the same direction of another finding on the protective role of ERI against racism effects using a child-report measure of racism [35]. It is possible larger and more representative samples that includes child-report measures of racism might yield even greater levels of poor SEWB and exposure to racism. Notwithstanding, our results reflect, at least partially, Aboriginal Australian children’s experiences of racism.

Another aspect to consider is that the ERI measure used in our study was not validated in the Australian Aboriginal context. Nevertheless, the items used to assess ERI reflect the Aboriginal community views on the importance of sense of belonging and pride to Aboriginal culture [24, 25]. LSIC measures and data collection procedures were conducted with extensive and ongoing community engagement. Across all waves, the LSIC study has involved close consultation with community stakeholders and therefore items such as ERI could be considered to be culturally appropriate [73]. Measures that can assist in identifying differences between the domains of process (e.g., exploration of the cultural practices and history of one’s ethnic-racial group) and content (e.g., feelings, beliefs, and the centrality of one’s ERI) in the development of Aboriginal children’s ERI might also assist in identifying specific variations in the relationship between their ERI and well-being [11, 19].

## Conclusions

The findings of the present study have a potential impact on public health. If ERI affirmation can modify the longitudinal effects of racism on child SEWB, promotion of a positive ERI can guide racial socialization practices, health communications, and integrate therapeutic approaches aimed to improve the mental health and well-being of Aboriginal Australian children [74]. The suggestion of the protective role of ERI affirmation is also relevant to mental health professionals’ clinical practice and interventions aimed to contribute to Aboriginal children’s positive development. The present study contributes to the evidence that ERI is a determinant of health and well-being to Aboriginal Australians. It corroborates the perspective that promotion of connection to culture and a positive regard towards being Aboriginal can assist in buffering the effects of risk factors, such as racism, in childhood. Future research should address specificities of the relationship between ERI components and SEWB in this population. Future studies will also be able to inform if increases in ERI over time contributes to improve SEWB and if its protective effects against risk exposure extends to adolescence and adulthood.
